# Brain functional connectivity differs when viewing pictures from natural and built environments using fMRI resting state analysis

**DOI:** 10.1038/s41598-021-83246-5

**Published:** 2021-02-18

**Authors:** Simone Kühn, Caroline Garcia Forlim, Anja Lender, Janina Wirtz, Jürgen Gallinat

**Affiliations:** 1 Lise Meiter Group for Environmental Neuroscience, Max Planck Institute for Human Development, Lentzeallee 94, 14195 Berlin, Germany; 2 University Clinic Hamburg-Eppendorf, Clinic and Policlinic for Psychiatry and Psychotherapy, Martinistraße 52, 20246 Hamburg, Germany; 3 Department of Psychology, Centre for Cognitive Neuroscience, Paris-Lodron-University of Salzburg, Hellbrunner Str. 34, 5020 Salzburg, Austria

**Keywords:** Human behaviour, Neuroscience

## Abstract

Human beings evolved in “natural” environments. Many intervention studies have shown that exposure to natural environments (compared to built/urban environments) reduces stress and increases cognitive functioning. We set out to test differences in fMRI functional connectivity while showing participants photographs from natural versus built environments (matched in terms of scenicness ratings). No differences in self-reported perceived stress, rumination, valence, arousal or dominance were observed. However, functional connectivity was significantly higher when participants saw natural rather than built environmental photographs in circuits consisting of dorsal attention network (DAN) and ventral attention network (VAN), DAN and default mode network (DMN) and DMN and Somatomotor connections. In addition, we observed lower functional connectivity during the natural environment condition correlated with more years that individuals spent in major cities during upbringing. Future studies, linking changes in cognitive functioning due to nature exposure and alterations in functional connectivity, are warranted.

## Introduction

Human beings evolved in what we would nowadays call “natural” environments. In contrast, urbanization is a comparably recent product of industrialization and therefore many evolutionary psychological theories have posited that humans have an innate tendency to seek out nature, e.g. the biophilia theory by Wilson^1^. And indeed, a multitude of intervention studies have demonstrated the positive effects that exposure to natural environment has on human beings (often times compared to exposure to built environments). On the one hand, exposure to natural environments is beneficial for cognitive functioning^2,3^, in particular on measures of attention and executive functioning. This seems to be true for real exposure but also for the exposure with digital representations of nature such as photographs, videos or virtual reality. On the other hand, exposure to natural environments, be it real or virtual, has been shown to lead to a reduction in arousal and stress^4^. The two main theories in the field, namely the attention restoration theory (ART) by Kaplan and Kaplan^5^ and the stress recovery theory (SRT) by Ulrich^6^ make attempts to explain these positive effects, with the first giving precedence to cognitive effects and the later to effects on stress. Upon closer inspection, both theories come to very similar predictions with a different assumption concerning the order of effects. However, a major gap in the present state of research is the lack of mechanistic knowledge on how the brain responds to exposure to natural and built environments, which is undoubtedly at the root of cognitive performance but also of stress responses. Knowing how the brain responds to natural versus urban environments constitutes a first step to potentially understanding how cognitive performance and arousal or stress is being altered as a consequence. Several studies using electroencephalography (EEG) have shown an increase in alpha activity when being exposed to natural stimuli^7–10^. Studies using functional magnetic resonance imaging (fMRI) have painted a fairly inhomogeneous picture of brain activation related to natural versus built stimuli^11–17^. Only two studies, that we are aware of, investigated brain connectivity: one examined fMRI in response to soundscapes of natural versus artificial sounds, observing increases and decreases in connectivity of a specific region of interest, but no global overall differences in connectivity^18^. Another study, using EEG, found more efficient and stronger brain connectivity with enhanced small-world properties in the nature versus urban real-life exposure^19^. From our point of view, a major drawback of previous studies focussing on brain activation is, that they neglected brain connectivity, which is a crucial characteristic of human brain function^20^. Measuring resting state functional connectivity captures “spontaneous” neural activity better, since it reflects a certain state^21^ and tonic rather than phasic processes. Another limitation of the previous studies investigating brain activity and brain connectivity during exposure to natural versus built/urban stimuli is the fact that in almost all studies the stimuli were not matched according to the dimension of perceived pleasantness. Commonly, natural environments are rated higher in pleasantness as well as aesthetics compared to built/urban environments. This phenomenon has been discussed previously, with some authors even stating that none of their natural stimuli showed pleasantness scores as low as the mean scores of the built stimuli^7,22^ and others simply reporting the significant differences in pleasantness ratings between their stimuli, e.g. Ref.^18^. Taken together, this constitutes a major confounding factor in these previous studies. Due to this confound, previous results cannot be unequivocally attributed to the differences in environment, but may instead—in part or fully—be driven by the difference in pleasantness/aesthetics. In order to address this concern, we employed a stimulus set consisting of photographs from natural versus built environments from Britain of whom we knew from an online rating study, that the average pleasantness ratings do not differ. With this stimulus set, we aimed to assess differences in whole-brain brain functional connectivity states using fMRI while passively viewing natural versus built environments. Because brain activation in response to single images was not the target of this study, a task-based fMRI paradigm was not used. Instead, we aimed at assessing state differences in resting state connectivity as previously introduced by Geerlings and colleagues^21^. After the separate exposure phases to pictures of natural versus built environment participants were asked several questions to assess stress, rumination, valence, arousal and dominance. We expected to find higher connectivity in the natural versus built environment condition within the default mode network based on the previous neuroimaging studies mentioned above and the SRT theory predicting higher relaxation.

## Methods

### Participants

Twenty-five healthy participants were recruited using online advertisements and flyers. After thorough description of the study, the participants’ informed written consent was obtained. The local psychological ethics committee of the University Medical Center Hamburg-Eppendorf, Germany, approved of the study. Participants had normal or corrected to normal vision (wearing contact lenses). The experiment was performed in accordance with relevant guidelines and regulations. Exclusion criteria for all participants were abnormalities in MRI, relevant general medical disorders and neurological diseases. Inclusion criteria were movement below the threshold of 0.5 mm^23^ during the scanning session and completion of all two conditions. After the inclusion and exclusion criteria were applied, the number of subjects dropped to 24 (mean age = 28.5, SD = 9.5 years, female:male = 16:8).

### Picture set

**Figure 1:**
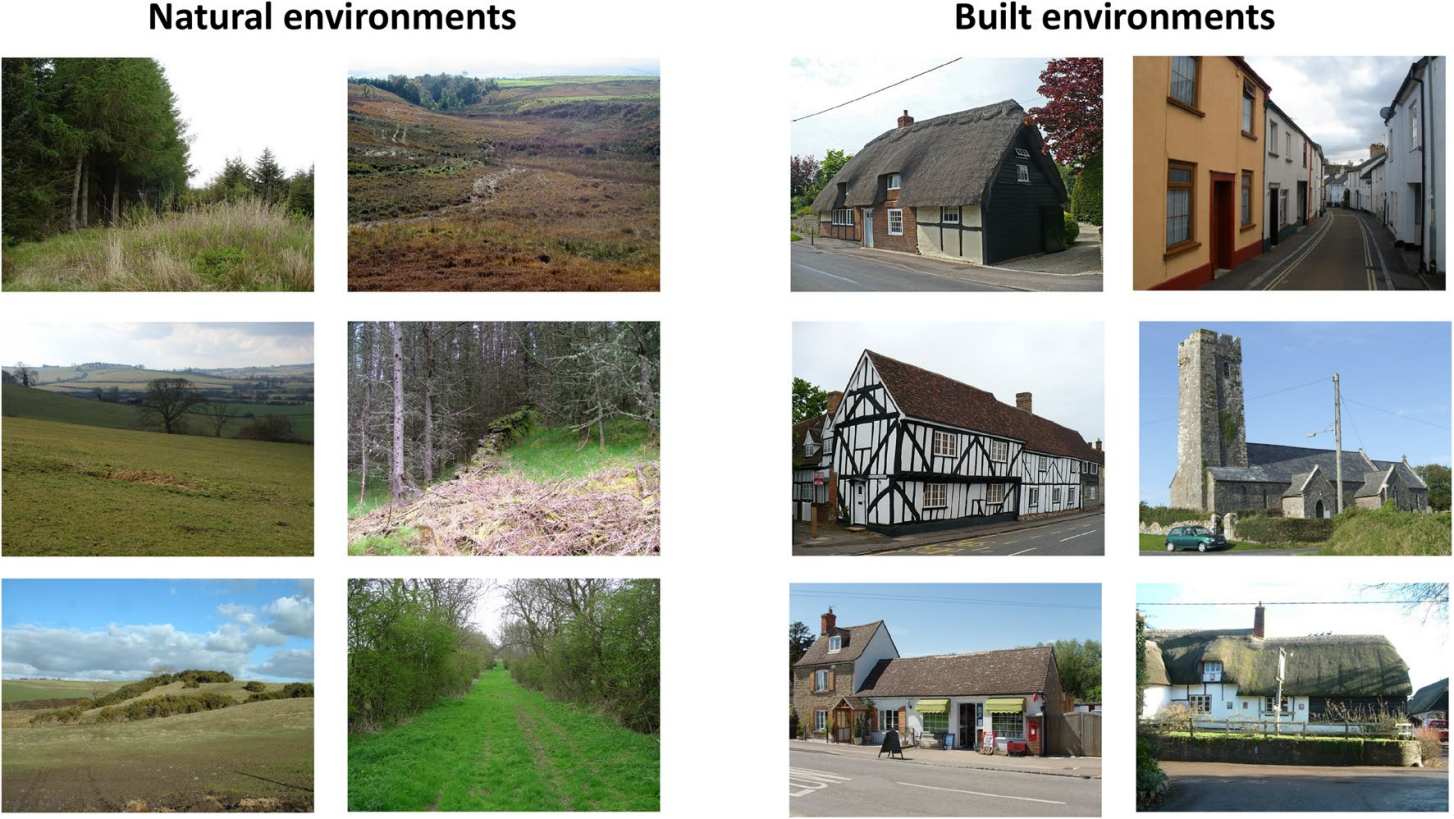
Example stimuli from the natural vs. built environment condition (photos taken from the website http://www.Geography.org.uk/). From left to right, top to bottom in each row: “Clocaenog forest” by Dot Potter CC, https://www.geograph.org.uk/photo/177009. “Hillside at Ashendon” by Andrew Smith CC. https://www.geograph.org.uk/photo/136564. “Gorse & Farmland near Endmoor” by David Medcalf CC. https://www.geograph.org.uk/photo/124098. “Looking down the Holm Burn” by David Robertson CC, https://www.geograph.org.uk/photo/578351. “Ruined Wall. Langamull Forest” by Mick Garratt CC, https://www.geograph.org.uk/photo/193780. “Catkill Lane” by Hugh Mortimer CC, https://www.geograph.org.uk/photo/161156. “Thatched Cottage, High Street” by Robin Drayton CC, https://www.geograph.org.uk/photo/832983. “Bunyan‘s Mead, Elstow” by Robin Drayton CC, https://www.geograph.org.uk/photo/824488. “Shop and post office, Stanton Harcourt” by David Hawgood CC, https://www.geograph.org.uk/photo/442708. “Clifford Street, Chudleigh” by Roger Cronfoot CC, https://www.geograph.org.uk/photo/1052835. “St Twynnells church” by Jonathan Billinger CC, https://www.geograph.org.uk/photo/521087. “The Royal Oak, Wootton Rivers” by Colin Bates CC, https://www.geograph.org.uk/photo/121152.

We selected 100 photographs taken in Great Britain, which were rated in terms of scenicness on the website http://scenicornot.datasciencelab.co.uk/ by means of crowdsourcing^24^. The website presents visitors with random photographs from the Geograph website, (http://www.Geography.org.uk/) an online documentation project encouraging users to submit geographically representative photographs of Great Britain. Each visitor can rate the pictures on a scale ranging from 1 to 10, where 10 indicates “very scenic” and 1 indicates “not scenic”. In total, over 1.5 million ratings have been submitted on that website for 217.000 photos. We selected images that were rated by more than 9 users (built condition: mean = 11.28, SD = 1.96; natural condition: mean = 11.40, SD = 1.70). Pictures with humans or animals were excluded. For the natural environment photographs (n = 50) an exclusion criterion was any man-made objects for the built environment photographs (n = 50) we searched for pictures showing buildings, but as little natural elements such as trees, lawn, or water as possible (Fig. 1). The average scenic rating of the photographs depicting natural environment was 5.061 (SD = 0.54) and the rating of the built environments was 5.062 (SD = 0.712) (*t*(98) = 0.007, *p* = 0.995). The number of votes did not differ between the natural and built pictures (*t*(98) = −  0.106, *p* = 0.916).

### Data acquisition

Structural images were collected on a Siemens Prisma 3T scanner (Erlangen, Germany) using a standard 32-channel head coil. The structural images were obtained using a three-dimensional T1-weighted magnetization prepared gradient-echo sequence (MPRAGE) (repetition time = 2500 ms; echo time = 2.12 ms; TI = 1100 ms, acquisition matrix = 240 × 241 × 194, flip angle = 9°; 0.8 × 0.8 × 0.94 mm voxel size). Functional data was acquired after the T1 image. We used a T2*-weighted echo-planar imaging (EPI) sequence (repetition time = 2000 ms, echo time = 30 ms, image matrix = 64 × 64, field of view = 216 mm, flip angle = 80°, slice thickness = 3.0 mm, distance factor = 20%, voxel size = 3 × 3 × 3 mm^3^, 36 axial slices, using GRAPPA). Images were aligned to the anterior–posterior commissure line (for similar description of methodology see Ref.^25,26^).

The data that support the findings of this study are available from the corresponding author upon reasonable request.

### Procedure

Within the scope of some demographic questions, we also asked how many years participants lived within major cities with more than 100.000 inhabitants during their first 15 years in life^27^.

After the questionnaire, participants underwent two functional scanning runs each lasting 5 min during which natural or built pictures were shown. The order was counterbalanced across participants. 50 pictures were shown in each scanning run. Each picture was displayed for 5 s and the inter-stimulus interval was 1 s. The images were projected via a mirror system. We refrained from introducing an attention checking procedure (e.g. button press demanded by a certain signal), since this may have disturbed the elicited state of brain activation that we were interested in investigating. Please note, that our conditions were neither designed for a task-based blocked design analysis, as we were not interested in which areas are activated during the conditions, nor in which condition evoked higher brain activation, but in comparing the brain states across the two conditions^21^.

After each condition, participants had to respond to several questions that were presented on the screen. Participants remained in the scanner for technical reasons and no functional images were acquired during this period. First, they were asked to answer five questions derived from the Perceived Stress Scale (PSS)^28^ (During the last 5 min, I…. felt nervous and “stressed”; …felt that things were going my way; …was “on top of things”; …was angered because of things that were outside of my control; …felt difficulties were piling up so high that I could not overcome them).

Then participants received three rumination related questions. They were measured by means of three items that have been used previously^29–31^. Participants were asked to rate how much the statements were applicable to them (During the past 5 min, I … could not get certain thoughts out of my mind; … I kept thinking about something over and over again; …had difficulties suppressing thoughts about myself).

Then three Self-Assessment-Manikins (SAM)^32,33^ illustrated in nine separate degrees were used to assess valence, arousal and dominance.

All items were responded to using a rating slider inside the MRI, ranging between the anchors “0” to “100”, which they operated with three buttons, one to move the pointer on the slider to the left, one to move it to the right and one to acknowledge and submit the response. When the “right” or “left” button was pressed down for longer the slider moved quicker in the respective direction. The order of questions was the same across all participants and both conditions. We decided to use the same slider for all questions, although this implied that we had to deviate from the previously validated response format, since our experience is that it is quite convenient for participants in the MRI scanner.

### Functional MRI preprocessing

The first 10 images were discarded due to steady-state longitudinal magnetization. First the data was corrected for slice timing and then realigned. Individual T1 images were co-registered to functional images and afterwards segmentation was performed into gray matter, white matter and cerebrospinal fluid. Data was spatially normalized to the MNI template. To improve signal-to-noise ratio, the data was spatially smoothed with 6-mm FWHM. To reduce physiological high-frequency respiratory and cardiac noise and low-frequency drift, filtering (0.01–0.09 Hz) was applied. Motion and signals from white matter and cerebrospinal fluid were regressed and the data detrended. Additionally, the voxel-specific mean framewise displacement (FD^23^) was computed. All subjects had FD values lower than the default threshold of 0.5 mm. FD per condition was: natural environment = (0.125 ± 0.076) mm and built environment = (0.136 ± 0.090) mm. All steps were executed in SPM12 (https://www.fil.ion.ucl.ac.uk/spm/) with exception of filtering, which was performed in REST toolbox^34^. All steps were done using MATLAB 2017a (www.mathworks.com) (for similar description of methodology see Refs.^25,26^).

### Functional connectivity networks

We first built the whole brain functional network, represented as a connectivity matrix, of each participant and then calculated the differences between the two conditions. How the connectivity matrices were built is described in the next paragraph. The statistical tool we used to compare the connectivity between conditions is described below in the section Statistical Analysis: network-based statistics (NBS).

*Connectivity matrices*: the connectivity matrices consist of nodes and edges. The nodes are regions of interest (ROI). We used ROIs from two brain templates: the automated anatomical labelling (AAL^35^) and the 7 functional network parcellation created by Yeo and colleagues^36^. Each brain template provides a parcellation with a different focus and therefore is able to unveil different aspects of information processing in the brain: AAL is a widely used brain parcellation based on anatomical regions resulting in 90 standardized regions whereas Yeo 7 network parcellation is based on intrinsic functional connectivity derived by means of cluster-based analysis of 1000 brains. The 7 resting state functional ROIs presented in Yeo atlas consist of: default mode network (DMN), Somatomotor, Dorsal Attention, Ventral Attention, Limbic, Frontoparietal and Visual networks.

**Figure 2:**
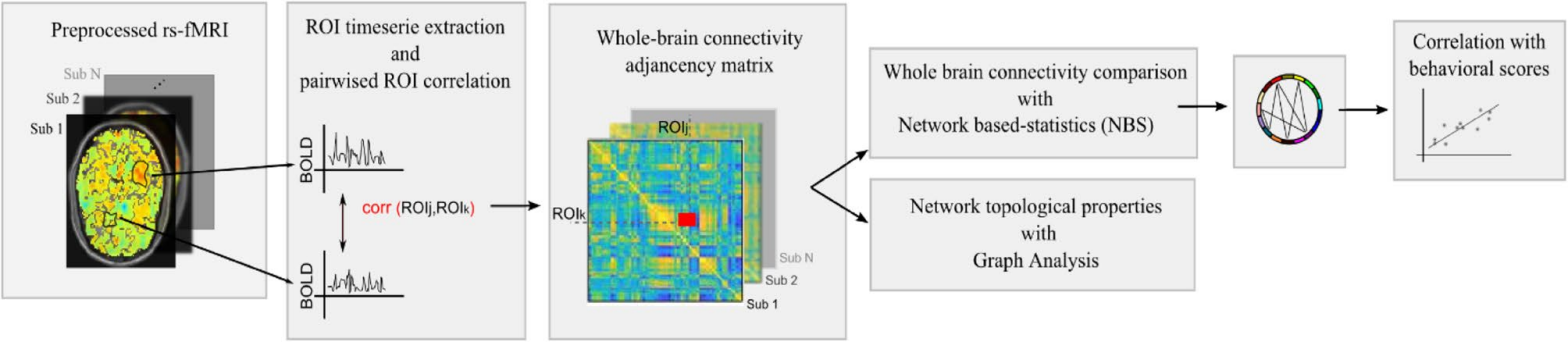
Diagram detailing the analysis pipeline.

The edges between the nodes represent the functional connectivity between two nodes. To calculate the functional connectivity between two nodes, first the timeseries of each ROI was extracted and then the Pearson correlation coefficient was calculated independently between each pair of ROIs (Fig. 2). The ROI timeseries were calculated based on the mean value of the pre-processed BOLD signal of all voxels within the respective ROI.

After the Pearson correlation coefficients were calculated independently for all pairs of ROIs, the resulting connectivity matrices from the AAL template were symmetric with a size of 90 by 90 (AAL has 90 ROIs and therefore 90 nodes) and the ones derived from the Yeo template were symmetric with a size of 7 by 7 (Yeo has 7 ROIs and therefore 7 nodes).

### Graph analysis

To complement the study of differences in functional connectivity between conditions, we opted to investigate the topological organization of the brain networks described above.

The topological organization of brain networks is of interest because it reflects how information can be exchanged in the network. Using graph analysis, the exchange of information is described based on concepts that characterize the networks as for example, hubs, clusters, shortest paths and small world among others. The specific graph measures applied here will be explained in the next paragraph.

Graph measures were calculated using the connectivity matrices derived from the AAL and Yeo brain parcellation (see above) using the Brain Connectivity Toolbox^37^. Graph measures are usually studied across a range of thresholds. The choice of these respective thresholds is arbitrary. In this study, self-connections were removed and the absolute values of the edges that surpass the threshold were set to 1 creating binary adjacency matrixes. We chose the proportional threshold method ensuring the same number of edges for all subjects in all conditions. The proportional threshold represents the number of edges present in the adjacency matrix divided by the total number of edges, and is referred to as the network cost K. We report a threshold range of 0.05 < K < 0.9 for the networks with nodes from the AAL template and 0.15 < K < 0.85 for the networks with nodes from Yeo template. The threshold step was 0.02. For each K within this range, the following measures were calculated per subject (for more details refer to Ref.^38^):

- Betweenness centrality (B): the fraction of all shortest paths that pass through a node,
- Transitivity (T): measuring the prevalence of clustered connectivity,
- Assortativity (A): a measure of network resilience that represents the correlation between the degrees of connected nodes,
- Global efficiency (Geff): the average inverse shortest path length,
- Small-worldness (S): characterized by simultaneously high cluster coefficient (C) and similar characteristic path length (L) as random networks; small world was calculated as: $S = \frac{{{\text{C}}/{\text{Crand}}}}{{{\text{L}}/{\text{Lrand}}}}$, $S \gg 1$. characterizes small-worldness, with Crand being the cluster coefficient of a random network and Lrand the characteristic path length of a random network.

In order to compute Crand and Lrand, a random network per subject is built that has similar features as the original network of the given subject; namely, the same number of nodes, mean degree and degree distribution. For that, four nodes were chosen at random (i_1_, j_1_, i_2_, j_2_) where node i_1_ was connected to node j_1_ (i_1_, j_1_) as well as node i_2_ was connected to node j_2_ (i_2_, j_2_). Then, the edges were rewired so that the edge between nodes (i_1_, j_1_) are “given” to nodes (i_1_, j_2_) and the edge between nodes (i_2_, j_2_) now belong to nodes (i_2_, j_1_) if the connection between (i_1_, j_2_) and (i_2_, j_1_) did not exist. In order to build a random network, this procedure was repeated multiple times (N = 10.000 for AAL template, N = 500 for Yeo template).

### Statistical analysis

#### Connectivity differences between conditions

To compare differences in connectivity between conditions we used Network based-statistics (NBS). NBS is a non-parametric statistical tool to compare brain networks^39^. NBS takes the connectivity matrices described above and identifies sets of connections (set of edges connecting nodes) that significantly differ between conditions while controlling for family-wise error (FWE). In short, a t-test is applied to every connection between nodes followed by a statistical threshold. In the remaining suprathreshold connections, a breadth-first search algorithm is used to obtain connected sets (sets of edges). The size of each set is given by their total number of connections. For each set of connections (set of edges), an FWE corrected p-value is estimated by permutation testing based on its size with a threshold of 5%. In this study, NBS was applied to the functional network matrices explained above in a paired t-test design with fixed parameters set to: N = 10,000, component size = extent and significance = 0.05. For the threshold we used values larger than 2. Mean FD was used as covariate. The analysis was performed by using the Network Based Statistic Toolbox v1.2 and MATLAB 2017a (www.mathworks.com). Results were visualized using the BrainNet Viewer^40^.

#### Correlation between connectivity and behavioural data

In order to investigate whether resting state functional connectivity in response to natural versus built environments was related to ratings after the respective condition and characteristics of the participants, the correlation between the mean connectivity of the set of connections from NBS analysis and these variables were calculated using Pearson correlation coefficient. In case of a significant correlation, an exploratory correlation analysis was conducted with individual connectivity value of each pair of nodes from the set of connections from NBS. The analyses were done using SPSS 25.

To investigate the correlation of the difference score in more depth, we split the variable “years of upbringing in major cities” into those ≤ 5 years and ≥ 10 years (excluding two subjects with intermediate values, namely 7 and 8 years) using a repeated measures ANOVA.

#### Graph analysis

The comparison of graph measures between conditions was done using nonparametric permutation test. For each graph measure and for each participant, we calculated the value of each graph measure at multiple thresholds, and then computed the area under the curve (AUC). The curve is given by the values of the graph measure across the different thresholds. Then we applied random permutation testing on the AUC values (n = 10.000) from which the *p* value was calculated. Correction for multiple comparison test due to the five graph measures was applied using False Discovery Rate (FDR) with significance set at *q* < 0.05.

## Results

### Ratings and questionnaire data

Most of the participants were living in a major city with more than 100.000 inhabitants, namely Hamburg, Germany, at the time of testing. On average participants lived 7.65 (SD = 6.29, range 0–15) years in major cities during their first 15 years in life.

The ratings after the natural and the built environment condition revealed no significant differences; neither in terms of perceived stress (*t*(22) = − 0.18, *p* = 0.86), nor rumination (*t*(22) = − 0.75, *p* = 0.46) or SAM (valence: *t*(22) = 1.62, *p* = 0.12; arousal: *t*(22) = − 1.08, *p* = 0.29; or dominance: *t*(22) = 0.92, *p* = 0.37).

### Functional connectivity

**Figure 3:**
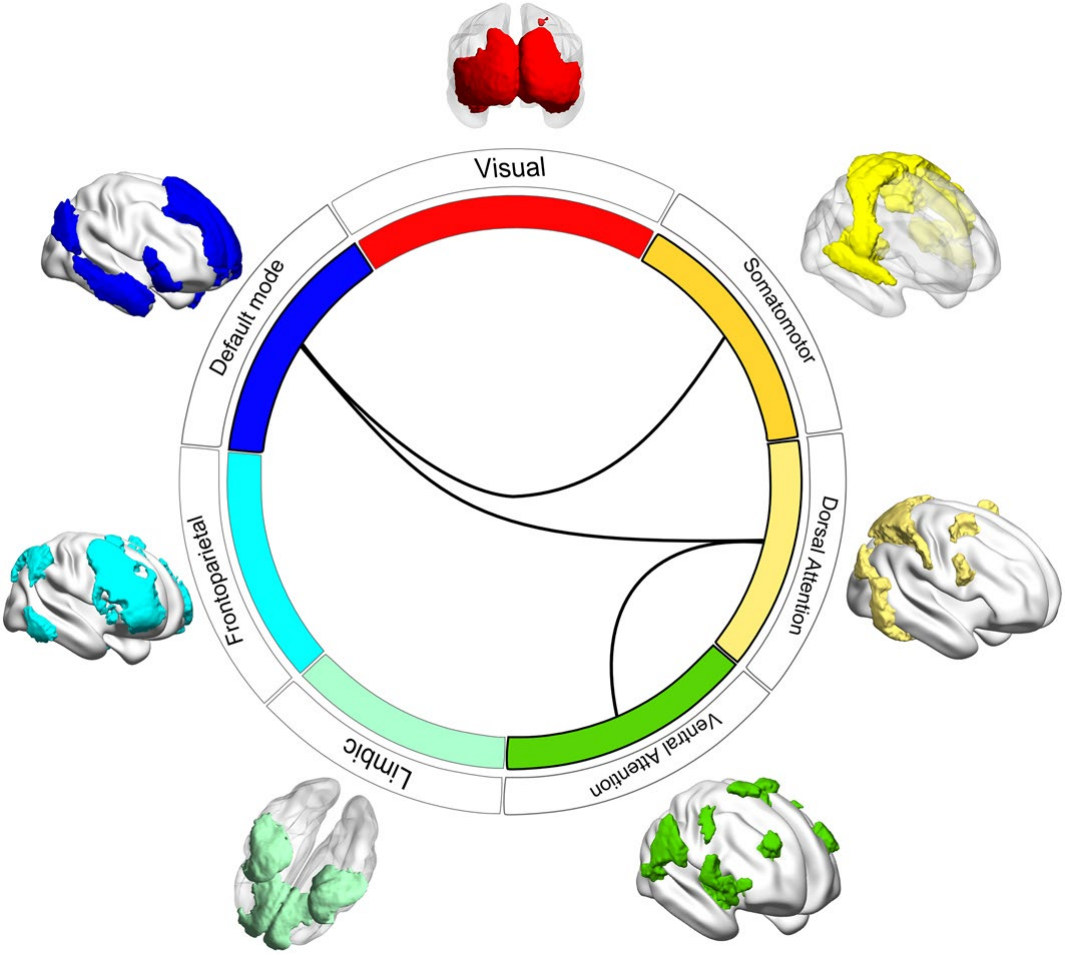
Set of connections from network-based statistics (NBS) showing higher functional network connectivity in response to natural as compared to built environmental stimuli. The different color bars represent the nodes, one per network of the Yeo template. The glass brain depicts the regions comprising each network. The lines represent the set of edges for which NBS identified differences in connectivity between conditions.

Differences in functional connectivity between natural and built environmental stimuli (paired t-test) were calculated using NBS statistical toolbox (Fig. 2). Differences in functional connectivity were observed in a set of connections calculated from the functional parcellation of the Yeo atlas (Fig. 3) (threshold = 2.5, *p* value = 0.01). The set of connections comprised the following functional ROIs (nodes in the connectivity matrix): default mode network (DMN), dorsal attention network (DAN), ventral attention (VAN) and the Somatomotor network (Fig. 3). In the condition with natural images, participants had higher functional connectivity between the nodes representing these networks, as compared to the condition where built environments were shown. More specifically, we observed an increase in connectivity between the nodes DMN and DAN, DMN and Somatomotor, and also between DAN and VAN. No differences in functional connectivity were found in brain networks calculated with the AAL template.

### Graph analysis

Graph analysis applied to whole brain networks represented as connectivity matrices derived from the AAL atlas (anatomical parcellation): We did not find differences in small-worldness properties between the two conditions of interest. Instead, we found higher values of transitivity (*p* = 0.029, uncorrected) and assortativity (*p* = 0.013, uncorrected) in natural as compared to built environments in functional networks with nodes from the anatomical brain parcellation of the AAL template (Supplementary Material Fig. S1a).

Graph analysis applied to whole brain networks represented as connectivity matrices derived from the Yeo atlas (functional parcellation): We found higher values of global efficiency (*p* = 0.048 uncorrected) in response to natural as compared to built environment exposure (Supplementary Material Fig. S1b). The results were not significant after correcting for multiple comparison (FDR, *q* > 0.05).

### Correlational analysis

**Figure 4:**
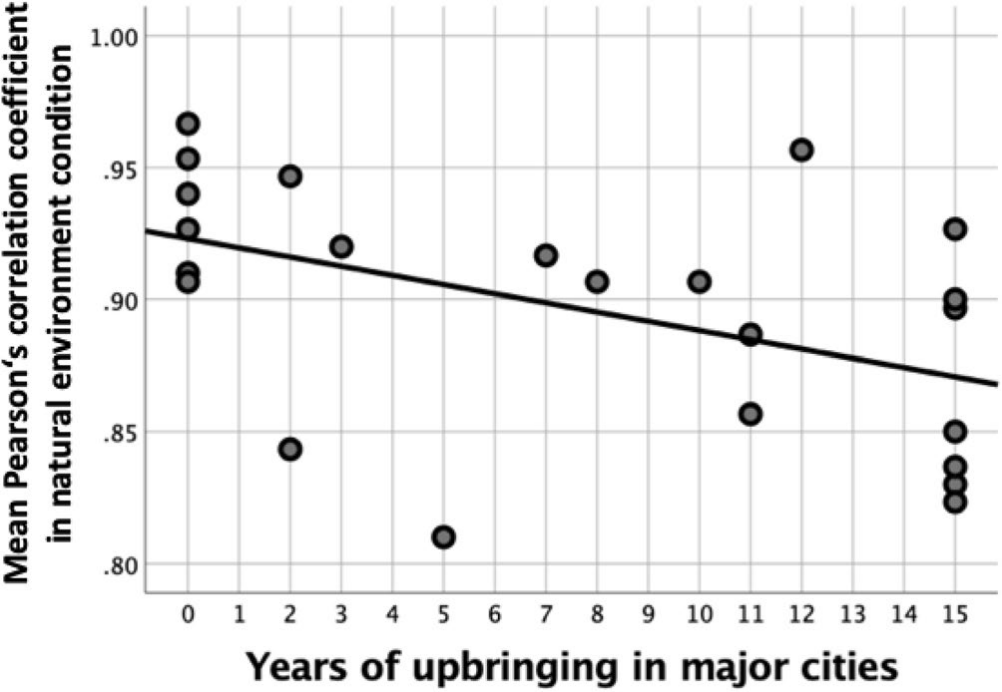
Scatterplots depicting the negative association between mean network connectivity (see Fig. 3) and years of upbringing in major cities.

The goal was to analyse whether the networks, in which we observed differences in connectivity between conditions (Fig. 3), were correlated with measures capturing inter-individual differences in upbringing. For that we calculated the average connectivity strength between the nodes corresponding to the DMN, DAN, VAN and Somatosensory network (Fig. 3), resulting in a mean functional connectivity network per subject. The mean functional connectivity network for the natural and the built environment condition were correlated with the amount of years spent in major cities during the first 15 years in life. We found a significant negative correlation between the mean connectivity strength during photos of natural environments and years of upbringing in major cities (*r*(23) = − 0.47, *p* = 0.022) (Fig. 4). The lower the connectivity strength, the more years during upbringing were spent in a major city. A post-hoc exploratory correlation analysis was conducted for each connection separately. We observed a significant negative correlation between years of upbringing in major cities and the connectivity strength between the nodes corresponding to the DMN and DAN in the natural environment condition (*r*(23) = − 0.53, *p* = 0.009, dark blue to light yellow in Fig. 3).

In order to ensure that the present results were not confounded by differences in global signal fluctuation (GSF) between participants we correlated years of upbringing in major cities with GSF and found no significant association (*r*(23) = 0.15, *p* = 0.52).

## Discussion

Within the scope of the present study, we set out to investigate potential differences in the brains’ functional connectivity at rest while showing participants photographs from natural versus built environments, since similar previous studies have mostly focussed on task-related brain activity instead. In contrast to previous studies, our stimuli were matched in terms of scenicness ratings, which ensures that the observed effects cannot be explained by differences in terms of aesthetics/pleasantness, as previous results have been^41^. The main finding of the present study was that functional network connectivity at rest was higher when participants were seeing natural in contrast to built environmental photographs in the nodes corresponding to the following networks: DAN and VAN, DAN and DMN and DMN and Somatomotor (Fig. 3). We found no differences in functional network connectivity for the reverse contrast. Interestingly, we found a negative association between functional connectivity in the natural environment condition and the years spent in major cities during the first 15 years in life. Concretely, the more time individuals spent in major cities the lower their functional resting state connectivity was within the aforementioned network (DAN, VAN, DMN and Somatomotor) while watching natural environmental photographs. When repeating the same analysis on scores representing the separate links of the network, the observed effect was strongest for the link between the DMN and DAN.

By focussing on brain functional connectivity instead of activity, this finding goes beyond previous studies, comparing brain activation in response to natural versus built pictures^11–17^, which yielded a very heterogeneous pattern of activation and deactivation. Another limitation of the previous studies is that most of the data was acquired in an Asian cultural context, which may not easily transfer to samples of Westerners due to differences in the environmental stimuli and the different cultural background of the participants. In contrast to the only previous fMRI study that focussed on brain connectivity, but presented soundscapes rather than photographs of natural and built environments^18^, we did observe global differences in functional brain connectivity. In the absence of reporting global connectivity differences between both conditions, the previous study reported connectivity of the posterior cingulate cortex (PCC), namely an increase of connectivity between PCC and precuneus and a decrease between PCC and medial prefrontal cortex when comparing the natural to the non-natural sound or a no-sound condition. In the present analysis these three regions, PCC, precuneus and medial prefrontal cortex were subsumed under the label of the DMN, because they are typically co-activated during intrinsic brain functioning and therefore treated in unity^36^. However, since we did not observe significant effects in functional connectivity when using the AAL atlas^35^, we can exclude that we would have been able to observe similar effects as Gould von Praag and colleagues using a more fine grained atlas in the present dataset. Previous results from a real-life nature versus urban exposure using EEG support this finding of more global connectivity differences^19^. Chen and colleagues report more efficient and stronger brain connectivity (in particular in the right hemisphere) with enhanced small-world properties within EEG signal recorded in situ in a natural compared with an urban environment. However, when explicitly running graph analyses on the present data set, with the goal to replicate this finding, we were unable to find an increase in small-world properties.

In terms of brain networks, which we found to be more strongly connected when seeing natural in contrast to built environmental photographs, it is remarkable that all connections, consisting of DAN and VAN, DAN and DMN and DMN and Somatomotor connections, were positively correlated. At first sight this seems to be at odds with one of the most influential findings in network neuroscience, namely the anticorrelation of networks, in particular of DMN and the DAN^42,43^. The idea was raised that the competition between DMN and “task positive” networks such as the DAN enable a toggling between internally and externally oriented cognitive processing^42^. However, more recent studies have refined this idea. A meta-analysis aimed at determining the strength of functional connectivity between DMN and DAN across multiple studies, found only weak negative correlations or even positive correlations (median effect size of *r* = − 0.06)^44^, suggesting a rather independent relationship between both networks. Interestingly, little stability of functional connectivity between DMN and DAN was observed across different cognitive contexts. However, due to a limited range of tasks, the authors were not able to specify principles on which cognitive states lead to which kind of relationship between DMN and DAN^44^. Another recent study came to the more specific conclusion that positive connectivity between DMN and DAN is associated with cognitive task performance (stop task vs. motor task vs. rest)^45^. While yet another study correlated cognitive performance with network brain connectivity and observed a positive association between DMN-DAN connectivity and speed tasks and a negative association with a fluid reasoning tasks for young adults (however, when controlling for age and including all participants the effects were non-significant)^46^. However, this finding is at odds with a study showing that attention leads to a de-correlation of connectivity between DMN and DAN, hence working against the intrinsically positive connectivity^47^ and an earlier study showing that the higher the anti-correlation between DMN and a task-related network was, the lower the reaction time variability in a cognitive performance task was^48^, indicating that a negative correlation between DMN and DAN is beneficial for cognition. In short, the previous evidence linking connectivity between DMN and DAN to attention and cognition in general is mixed. However, based on prior evidence, showing that different types of nature exposure (real walks, pictures, videos) lead to better cognitive performance and physiological relaxation after exposure^2–4^, we are tempted to interpret the increases in network connectivity observed in the present study as a positive effect.

The observation that the global connectivity strength within the networks depicted in Fig. 3 and in particular the strength of the link between DMN and DAN in the natural environment condition was negatively associated with years of upbringing in major cities, may hint at the long-term consequences of being raised apart from nature. It could be that city upbringing may potential reduce the beneficial properties of nature exposure. However, we cannot draw any causal conclusions concerning the direction of the effect. Previous research has associated early upbringing in major cities, during the first 15 years in life, with a 2.75 fold increased risk of schizophrenia^27^. This corresponds to the more widely known epidemiological findings that psychiatric diagnoses such as mood and anxiety disorders as well as schizophrenia are more frequent in urban compared to rural areas^49,50^. Previous neuroimaging evidence has revealed that urban upbringing is associated with a more pronounced activation in the dorsal anterior cingulate cortex (dACC) when undergoing a stressful math tasks in the MRI scanner^51^, a brain region that has been related to stress processing. The dACC is part of the VAN in the Yeo atlas we employed^36^, and part of the network identified to differ in terms of functional connectivity between viewing natural versus built environments in the present study. However, since we acquired data at rest without using any specific stress induction, the present study and the previously mentioned stress-exposure MRI study are difficult to compare. In general, dACC has been associated with a multitude of cognitive functions including cognitive control, monitoring, saliency processes, decision-making but also with emotional processes, as mentioned above^52,53^. Interestingly, recently it has been suggested that a unifying function in the contribution of dACC to the aforementioned cognitive processes might be that it represents contexts or task-state variables relevant for behaviour^54^.

Another series of studies on the link between environment and the brain has investigated associations between urban upbringing with brain structure. They consistently reported lower grey matter volume^55,56^ and thickness^57^ in dorsolateral prefrontal cortex (DLPFC) for individuals with a longer history of urbanicity exposure during upbringing. The DLPFC is part of the fronto-parietal network in the atlas we used, but in contrast to these previous brain structural findings does not appear involved in the network that we identified.

The graph analysis on the present data set revealed no significant results that survived multiple comparison correction. In particular, we did not find any differences in small-worldness as previously reported in a field-study, where more small-worldness was observed in the EEG signal recorded in a natural compared to an urban environment^19^. Since the methodology between this previous and the present study was clearly different (EEG vs. resting state fMRI), the setting was different (field experiment vs. photographs) and beauty matching differed (unmatched vs. careful matching of stimulus material) it is unclear which of the differences between the study presented here and this previous work explains the absence of effects.

### Limitations

A limitation of the present design is, that we did not assess cognitive functioning before and after the natural versus built environmental exposure. However, this was not possible due to time restrictions at the MRI scanner. In future studies the acquisition of changes in cognitive performance could provide a means to better estimate the functional relevance of the brain functional differences observed. Moreover, it might be helpful to collect physiological data during exposure in order to be able to interpret the observed functional connectivity differences to objectively assessed arousal levels and to link potential changes in cognitive performance to stress responses. Moreover, the present study overrepresented female participants, which makes the results less representative for males. In addition, the sample size was fairly small which may have in particular compromised the association between years of upbringing in major cities and the resting state connectivity measures. Here, future research is needed to confirm this finding. In the questions that we asked after the two conditions we used a slider which was not the previously validated response format. Future studies are needed to explore whether the validated response format is more successful in revealing associations between self-report scales and connectivity in response to exposure to natural versus built environments.

## Conclusion

To summarize, we investigated potential differences in fMRI functional connectivity at rest while showing participants photographs from natural versus built environments. In contrast to previous studies the stimuli used were matched in terms of scenicness ratings, which ensures that the observed effects are not confounded by differences in aesthetics/pleasantness. We observed no differences in self-reported perceived stress, rumination, valence, arousal or dominance between the two conditions. However, functional network connectivity at rest was higher when participants were seeing natural rather than built environmental photographs between the following networks: DAN and VAN, DAN and DMN and DMN and Somatomotor. No other connection between networks showed stronger functional connectivity during the built condition. In addition, we observed a negative association between functional network connectivity in the natural environment condition and the years spent in major cities during the first 15 years in life. Concretely, the more time individuals spent in major cities, the lower their functional resting state connectivity was within the aforementioned network (DAN, VAN, DMN and Somatomotor) while watching natural environmental photographs. When repeating the same analysis on the separate links of the network, the observed association with upbringing in major cities was strongest for the link between the DMN and DAN. Future studies, linking changes in cognitive functioning due to nature exposure and alterations in functional connectivity, are the obvious next step.

## Supplementary Information

Supplementary Information.
