# Supplementary material for: Brain functional connectivity differs when viewing pictures from natural and built environments using fMRI resting state analysis
Source: Sci Rep. 2021 Feb 18;11:4110. doi: 10.1038/s41598-021-83246-5 (PMC7893012; doi:10.1038/s41598-021-83246-5)

**Supplementary Material**

### **Brain functional connectivity differs when viewing pictures from natural and built environments using fMRI resting state analysis**

### Simone Kühn^1,2^, Caroline Garcia Forlim^2^, Anja Lender^2,3^, Janina Wirtz^2^, & Jürgen Gallinat^2^

^1^ Max Planck Institute for Human Development,

Lise Meiter Group for Environmental Neuroscience,

Lentzeallee 94, 14195 Berlin, Germany

^2^ University Clinic Hamburg-Eppendorf,

Clinic and Policlinic for Psychiatry and Psychotherapy,

Martinistraße 52, 20246 Hamburg, Germany

^3^ Paris-Lodron-University of Salzburg,

Department of Psychology, Centre for Cognitive Neuroscience,

Hellbrunner Str. 34, 5020 Salzburg, Austria

*Figure S1*: A. Graph analysis of the networks with nodes from anatomical parcellation (AAL). B. Graph analysis of the networks with nodes from functional parcellation (Yeo).

Figure S1:


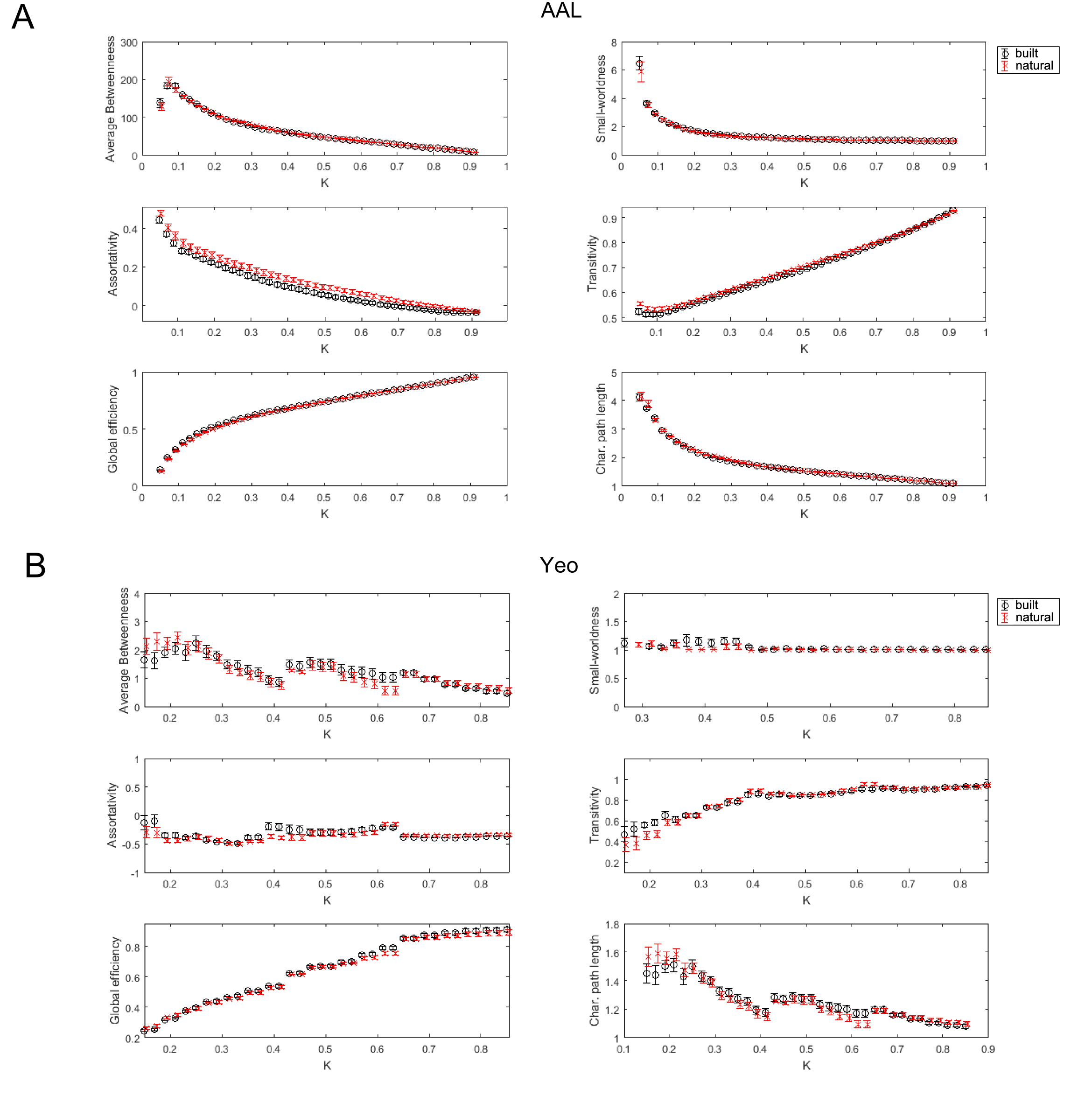

Supplement: Supplementary file 1 — Supplementary Information. [file 41598_2021_83246_MOESM1_ESM.docx]
